# Patient safety through quality assurance in in-vitro diagnostics – webinar 2024 of the Ad-hoc Commission on In-vitro Diagnostics of the Association of the Scientific Medical Societies in Germany (AWMF)

**DOI:** 10.3205/000341

**Published:** 2025-07-11

**Authors:** Michael Vogeser, Monika Brüggemann, Ulrich Sack, Albrecht Stenzinger, Henning Schliephake, Arbeitsgemeinschaft der Wissenschaftlichen Medizinischen Fachgesellschaften e.V.

**Affiliations:** 1 Institut für Laboratoriumsmedizin, Klinikum der Universität München, LMU München, Munich, Germany; 2 Klinik für Innere Medizin II, Sektion für Hämatologische Spezialdiagnostik, Universitätsklinikum Schleswig-Holstein, Campus Kiel, Germany; 3 Institut für Klinische Immunologie, Universitätsklinikum Leipzig, Germany; 4 Institut für Pathologie, Universitätsklinikum Heidelberg, Germany; 5 Klinik für Mund-, Kiefer- und Gesichtschirurgie, Georg-August-Universität Göttingen, Germany; 6 Berlin, Germany

## Abstract

The reliability of laboratory tests is ensured in particular by the interaction of regulated market access and certification of diagnostics, scientific evidence through guidelines, the guideline of the German Medical Association on quality assurance of laboratory medical examinations mentioned in § 9 of the German Medizinproduktebetreiberverordnung, as well as standardisation and accreditation. The Webinar 2024 of the Association of Scientific Medical Societies in Germany’s (AWMF) Ad-hoc Commission on In-vitro Diagnostics on 9 October 2024 highlighted this thematic network in four presentations from the perspective of the Federal Ministry of Health, the AWMF Institute for Medical Knowledge Management (IMWi), the German Accreditation Body (DAkkS) and the German Medical Association.

## Abstract (de)

Die Verlässlichkeit von Laboruntersuchungen wird insbesondere durch das Zusammenspiel von reguliertem Marktzugang und Zertifizierung von Diagnostika, wissenschaftlicher Evidenz durch Leitlinien, durch die in § 9 der Medizinprodukte-Betreiberverordnung adressierte Richtlinie der Bundesärztekammer zur Qualitätssicherung laboratoriumsmedizinischer Untersuchungen sowie durch Normung und Akkreditierung gewährleistet. Das Webinar 2024 der Ad-hoc-Kommission In-vitro-Diagnostik der Arbeitsgemeinschaft der Wissenschaftlichen Medizinischen Fachgesellschaften e.V. (AWMF) beleuchtete am 9. Oktober 2024 dieses thematische Netzwerk in vier Vorträgen aus Sicht des Bundesgesundheitsministeriums, des AWMF-Instituts für Medizinisches Wissensmanagement (IMWi), der Deutschen Akkreditierungsstelle (DAkkS) und der Bundesärztekammer.

## Webinar In-vitro Diagnostics 2024 of the Association of Scientific Medical Societies in Germany

Laboratory testing is fundamental to evidence-based healthcare; reliable laboratory results are a key determinant of patient safety in most clinical pathways. The Association of Scientific Medical Societies in Germany (AWMF) is dedicated to the continuous development of in-vitro diagnostics in the long term and pursues an interdisciplinary approach.

The reliability of laboratory tests is ensured in particular by the interaction of regulated market access and certification of diagnostics, scientific evidence through guidelines, the guideline of the German Medical Association on quality assurance of laboratory medical examinations addressed in Section 9 of the German Medical Devices Operator Ordinance, as well as standardisation and accreditation. The 2024 webinar of the AWMF Ad-hoc Commission on In-vitro Diagnostics highlighted this thematic network in four presentations on 9 October 2024.

In his presentation, **Steffen Buchholz** (Federal Ministry of Health, Division 124) dealt with EU Regulation 2024/1860 and the aim of reducing bureaucracy in the area of medical device and in-vitro diagnostics regulations. 

As an amending regulation, Regulation (EU) 2024/1860 of 13 June 2024 [1], [2] aims to ensure the availability of in-vitro diagnostic medical devices (IVDs). A central element is ensuring the availability of these diagnostics, which was ensured by revising the transitional provisions for products of various classes. Transitional periods during which IVDs can still be placed on the market under certain conditions on the basis of pre-existing IVD CE labelling have been defined as follows, depending on the product category:

- class D devices are granted a transitional period until 31 December 2027,
- class C devices will have a transitional period until 31 December 2028 and
- class B and A (sterile) devices will have a transitional period until 31 December 2029.

Another point is the gradual introduction of the EUDAMED database. This is intended to ensure transparency and product identification and help both authorities and the European Commission to obtain a comprehensive overview of the products on the EU market. The full implementation and mandatory use of EUDAMED was originally only to take place once all six modules of the database were fully functional. In order to accelerate the commissioning of already functioning modules, it has now been decided that the first individual modules will be introduced gradually, probably from the end of 2025.

The regulation also includes a reporting obligation for manufacturers in the event of supply bottlenecks. Manufacturers must notify the relevant authorities, distributors and healthcare providers at least six months in advance of an impending interruption in product availability. This is to ensure that there is sufficient time to take measures to minimise the risks to patients and public health. This reporting obligation comes into force on 25 January 2025.

In addition, Regulation 2024/1860 provides for the early, targeted evaluation of the Medical Device Regulation (MDR) and the In Vitro Diagnostic Medical Devices Regulation (IVDR) to review the effectiveness and feasibility of these regulations. Following structured consultations, a report by the EU Commission is planned for the end of 2025; this could potentially be followed by legislative measures. 

The other central topic of the presentation was a timely reduction in bureaucracy for the MDR IVDR. The political goal of this relief offensive is to reduce the bureaucratic burden by 25% without jeopardising the objectives of the regulations. In principle, the EU Commission plans to simplify reporting obligations and create legal certainty for companies, especially small and medium-sized enterprises (SMEs). To this end, a new SME and competition check is to be introduced for new legislative initiatives in order to assess the impact of new regulations on smaller companies. These measures are part of a larger plan by the EU Commission that focusses on reducing bureaucracy and simplifying implementation. Future legislation is also to be simplified specifically with regard to SMEs in order to promote their competitiveness. As part of these measures, the Commission wants to review existing legislation to ensure that it achieves its objectives and at the same time reduces the administrative burden. A reduction target of 35% of reporting obligations is envisaged for SMEs in particular. Another point is the increased use of digital solutions, which should enable faster and more efficient processing.

As a dialogue partner of the Federal Ministry of Health (BMG), the AWMF strongly supports initiatives to reduce bureaucracy in in-vitro diagnostics. This applies in particular with regard to in-vitro diagnostic medical devices that are manufactured in healthcare institutions exclusively for their own use. As these articles are not placed on the EU market, the AWMF recommends streamlining the relevant Article 5 (5) of the IVDR [3].

**Dr Monika Nothacker** (AWMF Institute for Medical Knowledge Management (IMWi)) gave a comprehensive overview of the AWMF Institute for Medical Knowledge Management (IMWi), the guideline register and current research projects on the digitalisation of medical knowledge.

The AWMF-IMWi was founded in 2009 and has offices in Marburg and Berlin. It supports the medical societies of the Association of the Scientific Medical Societies in Germany (AWMF) in the development and maintenance of guidelines. Its tasks include methodological support in the development and maintenance of the guideline register, the further development of guideline methodology and the organisation of training courses and consultations. In addition, the IMWi works on national and international projects that serve to ensure quality and improve medical care, for example as part of the oncology guideline programme and the National Health Care Guidelines (NVL).

Medical guidelines are systematically developed recommendations that reflect the current state of knowledge and support healthcare professionals and patients in their decision-making. These guidelines are based on the careful evaluation of scientific evidence and the weighing up of the benefits and harms of various treatment options. The aim is to provide guidance for practice, although deviations are possible in justified cases. In legal terms, guidelines are not binding standards, but rather recommendations; the medical standard is determined by experts in legal proceedings and is based on the state of scientific knowledge and best practice. Guidelines are not textbooks. They should address a relevant care problem when they are created.

The AWMF guidelines serve as a quality framework for the creation and evaluation of the guidelines listed in the guideline register. It was updated to version 2.0 in 2021 and to version 2.1 in 2023 and is based on international standards such as AGREE II and GRADE. This set of rules ensures that guidelines meet the high quality requirements and can be used as reliable decision-making aids in everyday medical practice. In order to promote the quality of guidelines, the IMWi provides methodological tools and instructions for systematic literature research and the evaluation of the evidence base of studies.

In the AWMF guideline register, guidelines are categorised according to their methodological approach: S1 guidelines: expert groups produce simple recommendations for action; S2 guidelines: based either on systematic evidence evaluation (S2e) or on the consensus of a representative panel (S2k); S3 guidelines: include both evidence assessment and consensus and are considered the highest quality guidelines.

The creation of an S3 guideline involves numerous steps, from the selection of topics, systematic literature research and formalised consensus procedures through to external review and publication. The aim is to ensure that guidelines reflect the current state of scientific knowledge and have practical relevance.

The IMWi is currently working on the ‘Dissolve-E’ project, which aims to digitise the AWMF guideline register. The aim of the project is to create an open, guideline-based and trustworthy evidence ecosystem. Digitisation should make guidelines more accessible and easier to integrate into everyday clinical practice in future, so that healthcare providers can access the latest recommendations at any time. Digitisation should also facilitate the long-term updating and expansion of the register and improve the availability of guideline knowledge.

The AWMF and the IMWi play a central role in the development, quality assurance and digitalisation of medical guidelines in Germany. The presentation highlights the importance of systematic and high-quality guidelines for medical care and the need to continuously develop and digitise them in order to make them accessible to all stakeholders in the healthcare system.

In his presentation, **Uwe Zimmermann** (Deutsche Akkreditierungsstelle GmbH) provided information on current developments and adjustments in the accreditation process for medical laboratories. The focus was on organisational changes, new processes at the German Accreditation Body (DAkkS) and international and digital developments.

The ‘Expert Council’ is a new body that is made up of former members of the sector committees, among others, and deals with technical issues. The aim of the Expert Council is to ensure the level of knowledge of the respective specialist areas and to integrate specialist expertise into the accreditation process. In addition, DAkkS replaces outdated documents with current standards (rules). It is not the task of DAkkS to define the state of the art in science and technology. This must be done by other organisations, e.g. scientific societies. Their checklists and assessment documents can still be used within the scope of accreditation, whereby deviations are only possible against the requirements of the underlying accreditation standard, possibly in conjunction with legal requirements.

Significant progress is seen in the introduction of the DAkkS portal, which was developed as part of the ‘Online Access Act’. The portal is intended to digitalise the entire accreditation process in the next stages, which should result in increased efficiency, a reduction in bureaucracy and improved application processing. Applications can now be submitted and tracked digitally. The portal also allows the digital exchange of documents and formal correspondence between DAkkS and the accredited bodies, which contributes to communication without media discontinuity. It is free of charge for all accredited bodies and is intended to increase user-friendliness and transparency in the accreditation process.

Another part of digitalisation is the digital accreditation symbol, the so-called eAttestation. This system enables a machine-readable, tamper-proof and electronically verifiable representation of the accreditation and the digital results reports. It ensures the authenticity and integrity of the reports and is intended to establish a digital quality infrastructure that enables customers and authorities to digitally verify the status of accreditation. Applications to use this digital symbol can be submitted from April 2024.

The ISO 15189 standard, which specifies requirements for the quality and competence of medical laboratories, was published in a slightly modified German version in August 2024. The changes include an adaptation of translations and editorial clarifications as well as the addition of the informative Annex ZA. This establishes the link between the standard and the legal requirements – in this case Regulation (EC) No. 765/2008. Accreditations based on DIN EN ISO 15189:2023 remain valid and are equivalent, as the 2024 version in the normative part is merely an updated translation of ISO 15189:2022. The DAkkS informed affected laboratories that they do not need a new assessment for the change from version 2023 to version 2024.

The European Accreditation (EA) and the International Laboratory Accreditation Cooperation (ILAC) have published new regulations on the accreditation of flexible scopes. Since April 2020, the EA has required laboratories with flexible accreditation to make an up-to-date list of accredited procedures publicly available. This serves the purpose of transparency and helps to disclose the full scope of an accreditation to all relevant stakeholders. For Germany, this regulation is based on the Accreditation Bodies Act (AkkStelleG), which ensures that accreditation information is always up-to-date and publicly available.

The presentation explains that DAkkS has taken comprehensive measures to increase efficiency and digitalise the accreditation process. The introduction of the DAkkS portal, the digital accreditation symbol and the adaptation of ISO 15189 are steps that are intended to create a modern and user-friendly accreditation landscape. International guidelines on transparency and flexibilisation are being integrated into the German accreditation process, which will increase the quality and transparency of medical laboratories in Germany in the long term.

The AWMF emphasises the importance of close professional cooperation between the scientific societies within the AWMF and the DAkkS in determining and formulating the state of the art in science and technology in the respective subject areas.

In his presentation, **Alexander Golfier** (German Medical Association – BÄK) addressed the role and limits of standards in medicine. In his presentation, he emphasised the high relevance of technical standards for medical devices, including in-vitro diagnostics, for patient safety and pointed out the problematic application of standards for healthcare services and, in particular, for medical activities.

Technical standards ensure reliable in-vitro diagnostics and other medical devices and safe medical procedures in these areas and are therefore indispensable. To this end, the BÄK has long been actively involved in the technical standardisation process both nationally (German Institute for Standardisation – DIN) and internationally (CEN and ISO). In the DIN Standards Committee Health Technologies – NA GesuTech (merged from the NA Medicine and the Standards Committee Rescue Service and Hospital), the BÄK provides the deputy chairman, Mr Golfier.

However, the BÄK has major reservations regarding the standardisation of healthcare services and categorically rejects the standardisation of medical activities, e.g. DIN EN 16372 Aesthetic surgery services [4]. 

From the point of view of the medical profession, standards are neither a necessary nor a suitable instrument for the area of healthcare services and in particular for the complex area of original medical activities, through which the quality of service provision could be ensured or improved. The criticism of the standardisation of healthcare services and in particular medical activities was already set out in detail in 2015 in a statement by the German Medical Association.

Criticism of the standardisation of healthcare services is supported by practically all organisations and associations relevant to the healthcare sector, e.g. (but by no means exhaustively) the umbrella organisations of the German Social Insurance, the Federal Ministry of Health (BMG), the German Medical Association, the National Association of Statutory Health Insurance Physicians, the Confederation of Liberal Professions, the European Trade Union Confederation (ETUC), the Standing Committee of European Doctors (CPME) and the World Medical Association.

In Germany, the DIN Standards Committee for Services (NADL) is responsible for service standards. For this reason, the BÄK has also been involved in this important DIN committee for years and currently has Mr Golfier as NADL chairman. 

The application of standards is basically voluntary. However, the application of standards can be stipulated as binding in private law contracts, in government regulations or by other means. A special category of EU standards are ‘harmonised standards’, which are commissioned and financed by the European Commission. Companies that apply such harmonised standards are presumed to meet the technical requirements stipulated by the EU. Nevertheless, the application of harmonised standards is also voluntary.

Standardisation offers not only safety-related but also economic benefits. For Germany, based on the period 2002 to 2006, the overall economic benefit of standardisation is estimated at around 17 billion euros per year. The annual benefit is now likely to be significantly higher. Companies benefit not only from the safety and quality standards set by standards, but also from the opportunity to introduce their own technologies into standardisation processes and thus help shape the market. Standards fundamentally strengthen international trade and are categorised as the language of world trade.

In addition to the standards, the standardisation product ‘DIN SPEC’ will also be presented, which is becoming increasingly important in the standardisation process. According to DIN, a DIN SPEC standard can be easily developed within a few months and then published nationally and/or internationally by DIN. If DIN SPEC standards are successful, they can serve as the basis for a standardisation project. However, there is a risk that a DIN SPEC – as is the case with DIN SPEC 91460 ‘Delirium management in inpatient facilities’ from the perspective of the BÄK, the KBV and the Association of the Scientific Medical Societies in Germany (AWMF) – may be flawed in rare cases. The BÄK, the KBV and the AWMF had already rejected the transfer to a DIN SPEC 91460 standard in advance. In principle, however, DIN SPEC standards are certainly to be welcomed, as innovative product or service ideas can be implemented quickly.

The AWMF also shares the view that technical standards for medical devices/in-vitro diagnostic products make a major contribution to quality assurance. The standardisation of healthcare services/medical activities, on the other hand, is also associated with considerable risks in the view of the AWMF, as they cannot meet the dynamic and individual requirements of medical practice. Standards in the medical context must be considered in a differentiated manner and may only be applied where they do not stand in the way of the complex requirements of medical practice.

The webinar took place on 9 October 2024 and was followed by around 170 participants. The presentation slides of all contributions can be viewed on the AWMF website (https://www.awmf.org/die-awmf/veranstaltungen/symposien).

## Notes

### Competing interests

The authors declare that they have no competing interests.
